# Prognostic value of CALLY index in combination with the GRACE score among patients with acute myocardial infarction: a retrospective cohort study

**DOI:** 10.3389/fcvm.2026.1858524

**Published:** 2026-09-18

**Authors:** Chuanglu Zhao, Rong Chen, Ruirui Feng, Xianlin Xu, Hang Sun, Xinwen Min, Handong Yang, Jishun Chen, Xiaolei Li

**Affiliations:** 1 Sinopharm Dongfeng General Hospital (Hubei Clinical Research Center of Hypertension), Hubei University of Medicine, Shiyan, Hubei, China; 2 Sinopharm Dongfeng Huaguo Hospital, Shiyan, Hubei, China

**Keywords:** acute myocardial infarction, CALLY, GRACE, MACCE, prognosis

## Abstract

**Objective:**

The study aimed to investigate the prognostic value of the CRP-albumin-lymphocyte (CALLY) index for adverse outcomes in patients with AMI and evaluate the potential value of combining the CALLY index with the GRACE score to improve prognosis.

**Methods:**

This study enrolled 1,127 patients with acute myocardial infarction (AMI) admitted to the Sinopharm Dongfeng General Hospital from 2021 to 2023. Patients were stratified into two cohorts based on the median CALLY index score. Kaplan–Meier survival analysis, multivariable Cox proportional hazards models, and restricted cubic spline (RCS) analysis were used to assess the association between CALLY index levels and major adverse cardiovascular and cerebrovascular events (MACCE). Time-dependent receiver operating characteristic (Time-ROC) curves were constructed to evaluate the prognostic value of the CALLY index for AMI patients. Additionally, subgroup analyses were conducted to assess the robustness and consistency of this association across different clinical characteristics.

**Results:**

During a median follow - up of 24 months, a total of 243 patients (24%) experienced a major adverse cardiovascular event (MACCE). The CALLY index in the MACCE group was significantly lower than that in the non - MACCE group. In the fully adjusted model, whether treated as a continuous variable (HR = 0.68; 95% CI: 0.51–0.90) or as a categorical variable (HR = 0.29; 95% CI: 0.19–0.44; *P* < 0.001), a lower CALLY index was associated with an increased risk of MACCE in patients with AMI. RCS analysis revealed a nonlinear relationship between the CALLY index and prognosis in AMI patients (nonlinear *P* - value < 0.001). ROC curve analysis indicated that the CALLY index has good predictive ability for MACCE (AUC = 0.788; 95% CI: 0.742–0.835), and that combining the CALLY index with the GRACE score significantly improves predictive ability. In subgroup analyses, the CALLY index demonstrated good generalizability and robustness.

**Conclusion:**

The CALLY index was closely associated with patient prognosis in acute myocardial infarction (AMI) and significantly enhanced the predictive accuracy of the traditional GRACE score. The CALLY index was poised to emerge as a novel biomarker for prognostic stratification in AMI.

## Introduction

Acute myocardial infarction (AMI) ranks as a top cause of mortality due to cardiovascular disease, with atherosclerosis (AS) being a key and most critical root cause. A heightened inflammatory reaction significantly contributed to the emergence and advancement of atherosclerosis (1, 2). The incidence and mortality rates of AMI continued to rise. Data suggests that by 2024, the worldwide count of acute myocardial infarction (AMI) patients is projected to hit around 64 million, with up to 60% of these cases being ST-segment elevation myocardial infarction (STEMI), presenting a significant risk to human health. The prompt detection of significant cardiovascular incidents MACE), whether occurring inside or outside the hospital, has emerged as a formidable hurdle in medical procedures (3, 4).

The Global Registry of Acute Coronary Events (GRACE) score was acknowledged as an effective predictor for acute coronary syndrome (ACS) (5). Subsequent to ongoing enhancement and confirmation, research has shown that the GRACE score is significantly predictive of both the six-month mortality risk post-discharge and short-term combined incidents, including death and myocardial infarction (6, 7). Lately, various researches have integrated new biomarkers into the GRACE score to improve its forecasting accuracy. As an example, the high-sensitivity C-reactive protein (hs-CRP) showed a notable positive link with the GRACE score, and incorporating it into the scoring system enhanced its predictive accuracy for negative clinical results (8). Additionally, the evolving alterations in BNP have been confirmed to be crucial in enhancing the predictive accuracy of the GRACE score, as ongoing observation aids in the precise pinpointing of patients at high risk (9).

The CRP-Albumin-Lymphocyte (CALLY) index, as a novel inflammation-immunity-nutrition scoring system, comprehensively reflects the body's inflammatory and immune status and is associated with the prognosis of various cardiovascular diseases (10–13). Previous studies have demonstrated that a high CALLY index has a protective effect against both short and long-term MACCE in patients with STEMI, highlighting the significance of inflammatory immunity in the AMI population (14). Additionally, the CALLY index was closely associated with the occurrence of the no-reflow phenomenon following percutaneous coronary intervention (PCI) in AMI patients. It helps clinicians predict this complication and further improves treatment decision-making (15, 16).

As a novel inflammatory marker, the CALLY index has not yet been fully studied in terms of its prognostic value for AMI or its potential to enhance the predictive ability of the GRACE. Therefore, this study aims to investigate the association between the CALLY index and the incidence of MACCE in AMI, and to analyze the incremental value of the CALLY index to the GRACE. The goal was to provide a new reference dimension for risk stratification in AMI patients.

## Methods

### Study population

The research conducted a retrospective examination of 1,127 individuals identified with Acute Myocardial Infarction (AMI) at the Heart Center of Guoyao Dongfeng General Hospital between January 2021 and December 2023. Detailed collection of cardiovascular risk factors, demographic information, historical medical records, lab data, test outcomes, and medication usage was conducted on medical cases. Inclusion Criteria: Diagnosis of AMI was based on the Fourth Universal Definition of Myocardial Infarction. The following conditions are considered as acute myocardial infarction: at least one value above the 99th percentile URL and at least one of the following: symptoms of myocardial ischaemia; new ischaemic ECG changes; development of pathological Q waves; imaging evidence of new loss of viable myocardium or new regional wall motion abnormality in a pattern consistent with an ischaemic aetiology; identification of a coronary thrombus by angiography or autopsy. And the exclusion criteria were: (1) Age under 18 years; (2) Severe liver dysfunction (transaminase > 5 times the upper limit of normal); (3) Severe renal failure (estimated glomerular filtration rate [eGFR] < 30 mL/min/1.73m^2^ or undergoing renal replacement therapy); (4) Malignancy or severe autoimmune disease; (5) Severely incomplete clinical data; (6) Lost follow-up. A total of 1,010 patients with AMI were included in the final analysis (Figure 1).

**Figure 1:**
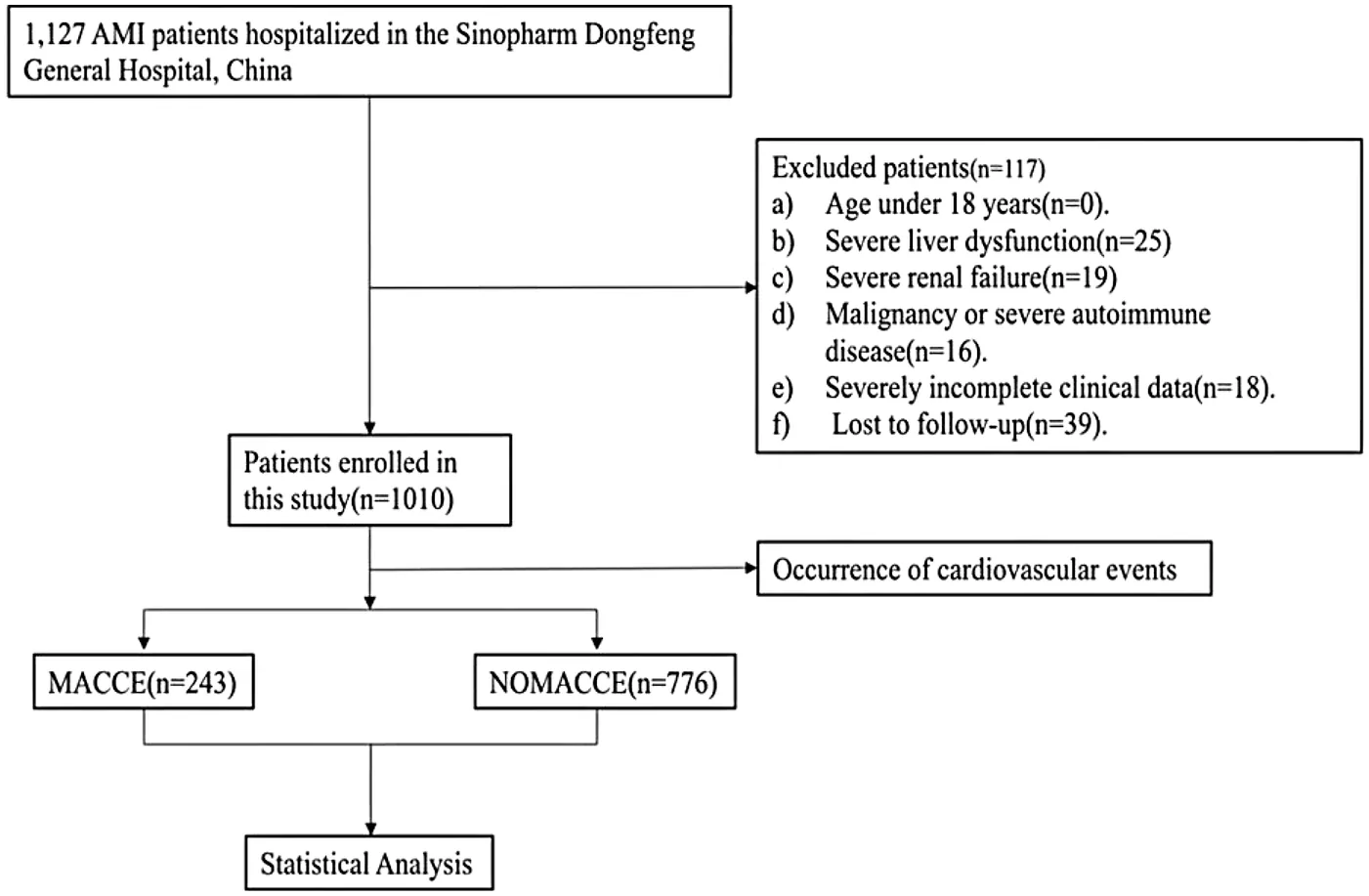
Flowchart of the study population.

### Endpoint and definitions

The primary endpoint of this study was major adverse cardiovascular and cerebrovascular events (MACCE), including cardiac death, non-cardiac death, non-fatal myocardial infarction, target vessel revascularization, heart failure, and non-fatal stroke. Meanwhile, all patients were followed for at least one year or until the endpoint event occurred.

The specific definitions were as follows:

Cardiac Death: Cardiac death was defined as death caused by cardiovascular diseases, including acute myocardial infarction (MI), sudden cardiac death, heart failure, stroke, cardiovascular surgery, cardiovascular hemorrhage, and death resulting from other cardiovascular causes. Non-cardiac Death: Non-cardiac death was defined as any death not considered to be of cardiovascular origin, including deaths caused by malignancy, pulmonary causes, infection, gastrointestinal causes, accidents or trauma, non-cardiovascular organ failure, and other non-cardiovascular causes. Death of Undetermined Cause: Death of undetermined cause was defined as any death that could not be classified into other categories due to the absence of relevant evidence. Non-fatal Myocardial Infarction (MI):Non-fatal MI was defined based on the time elapsed since the procedure: Postoperative > 48 h: Defined as an elevation of either cardiac troponin I or T (cTnI/cTnT) greater than 1 times the upper reference limit (URL), accompanied by at least one of the following: symptoms of acute myocardial ischemia, new ischemic electrocardiographic (ECG) changes, development of pathological Q waves, imaging evidence of new loss of viable myocardium or new regional wall motion abnormality, or identification of a coronary thrombus via angiography, intracoronary imaging, or autopsy. Postoperative < 48 h: For patients with normal preoperative card iac troponin levels: Defined as an elevation of either cTnI/cTnT greater than 5 times the URL. For patients with elevated but stable (fluctuation within 20%) or falling preoperative cardiac troponin levels: Defined as an elevation of either cTnI/cTnT greater than 5 times the URL *and* an increase of ≥20%. In addition, all patients in this category were required to exhibit at least one of the following: symptoms of acute myocardial ischemia, new ischemic ECG changes, development of new pathological Q waves, imaging evidence of new loss of viable myocardium or new regional wall motion abnormality, or angiographic findings consistent with a procedural complication limiting coronary blood flow. Target Vessel Revascularization (TVR): Target vessel revascularization was defined as any repeat percutaneous intervention or surgical bypass performed on the target vessel. Non-fatal Stroke: Non-fatal stroke was defined as the sudden onset of neurological deficit symptoms, after the exclusion of head trauma, which was confirmed by head imaging showing hemorrhage or infarction in the brain region corresponding to the symptoms. The CALLY index was calculated using the following formula: CALLY index = (Albumin × Lymphocyte count)/(CRP × 10). The GRACE score was calculated for each patient upon admission using the online GRACE calculator. The GRACE risk criteria included age, heart rate, systolic blood pressure, initial serum creatinine, Killip class, cardiac arrest upon admission, troponin I level, and ST-segment changes.

The study was carried out in accordance with the ethical principles outlined in the Helsinki Declaration and approved by the Ethics Committee of Sinopharm Dongfeng General Hospital (Approval No. LW-2024-056). Given the retrospective design of the study, the review board waived the requirement for informed consent. The study protocol was registered and approved by the Chinese Clinical Trial Registry (Approval No. ChiCTR2500095817).

### Statistical analysis

Statistical analyses were performed using SPSS version 26.0 and R version 4.1. Continuous, normally distributed variables were described as mean ± standard deviation and compared with the t-test; non-normally distributed variables were summarized as median (P25, P75) and compared with the Mann–Whitney U-test. Categorical variables were expressed as frequency (percentage) and compared using the Chi-square test or Fisher's exact test, as appropriate. The Kaplan–Meier method was applied to construct cumulative survival curves, and differences between groups were compared using the Log-Rank test. Univariate and multivariate Cox proportional hazards models were used to examine the association between CALLY and MACCE. Restricted cubic splines (RCS) were employed to characterize the dose-response relationship between CALLY and major endpoint events. Receiver operating characteristic (ROC) curves were plotted to indicate the predictive value of CALLY for MACCE. All tests were twosided, and a significance level of *P* < 0.05 was considered statistically significant.

## Results

### Baseline characteristics of patients in different prognostic groups

During a median follow-up of 24 months, 243 (24%) patients experienced MACCE; detailed data are presented in Supplementary Table S1. As shown in Table 1, compared with the non-MACCE group, patients in the MACCE group were older, included a lower proportion of male patients, had higher heart rates, and showed a higher prevalence of hypertension and diabetes (all *P* < 0.05). Furthermore, the MACCE group exhibited lower levels of hemoglobin, lymphocyte count, albumin, LVEF, and CALLY index, whereas levels of glycosylated hemoglobin, C-reactive protein, creatinine, total cholesterol, GRACE score, and NT-proBNP were elevated (all *P* < 0.01). Regarding therapeutic regimens, patients in the MACCE group received conservative therapy more frequently, with lower utilization rates of P2Y12 receptor inhibitors, aspirin, ACEi/ARB/ARNI, and beta-blockers, whereas diuretic use was higher.

**Table 1:** Baseline clinical characteristics of patients stratified by MACCE.

Variables	Total (*n* = 1010)	non-MACCE (*n* = 767)	MACCE (*n* = 243)	*P*
Age (year)	67.50 (57.00, 75.00)	66.00 (56.00, 74.00)	72.00 (61.50, 78.50)	<.001
Male, *n* (%)	698 (69.11)	543 (70.80)	155 (63.79)	0.039
BMI (kg/m^2^)	23.44 (21.20, 24.60)	23.44 (21.20, 24.61)	22.49 (21.20, 24.03)	0.019
SBP (mmHg)	132.00 (117.00, 148.00)	132.00 (116.00, 147.50)	135.00 (117.50, 153.00)	0.167
DBP (mmHg)	77.00 (69.00, 89.00)	77.00 (69.00, 88.00)	78.00 (69.00, 89.00)	0.404
Heart rate (bpm)	78.00 (68.00, 90.00)	77.00 (68.00, 89.00)	79.00 (70.00, 92.50)	0.036
Smoking, *n* (%)	453 (44.85)	361 (47.07)	92 (37.86)	0.012
Drinking, *n* (%)	180 (17.82)	141 (18.38)	39 (16.05)	0.407
Hypertension, *n* (%)	601 (59.50)	436 (56.84)	165 (67.90)	0.002
Diabetes, *n* (%)	244 (24.16)	164 (21.38)	80 (32.92)	<.001
Stroke, *n* (%)	56 (5.54)	42 (5.48)	14 (5.76)	0.865
WBC (10^9^/L)	7.80 (6.18, 9.94)	7.86 (6.18, 9.89)	7.73 (6.18, 10.25)	0.927
NEUT (10^9^/L)	5.77 (4.10, 7.88)	5.77 (4.10, 7.80)	5.86 (4.19, 8.46)	0.437
LYMPH (10^9^/L)	1.33 (0.91, 1.80)	1.38 (0.97, 1.81)	1.20 (0.80, 1.65)	0.001
Hb (g/L)	134.00 (119.00, 147.00)	136.00 (120.00, 148.00)	129.00 (112.50, 144.50)	<.001
HbA1c (%)	5.90 (5.50, 6.70)	5.90 (5.50, 6.60)	6.10 (5.60, 7.43)	0.002
CRP (mg/L)	5.28 (1.52, 18.43)	4.30 (1.23, 15.62)	8.16 (3.05, 24.73)	<.001
K^+^ (mmol/L)	3.80 (3.50, 4.10)	3.75 (3.50, 4.10)	3.80 (3.60, 4.10)	0.044
Na^+^ (mmol/L)	139.00 (137.00, 141.00)	139.00 (137.00, 141.00)	139.00 (137.00, 141.00)	0.678
Cl^+^ (mmol/L)	107.00 (104.00, 109.00)	107.00 (105.00, 109.00)	107.00 (103.50, 109.00)	0.096
Ca^2+^ (mmol/L)	2.20 (2.12, 2.28)	2.21 (2.12, 2.29)	2.18 (2.11, 2.26)	0.032
Urea (mmol/L)	5.66 (4.45, 7.39)	5.56 (4.34, 7.08)	6.31 (4.74, 9.73)	<.001
Crea (umol/L)	73.00 (62.00, 97.75)	72.00 (61.00, 89.00)	86.00 (64.00, 121.00)	<.001
Glu (mmol/L)	7.30 (5.90, 9.50)	7.20 (5.90, 9.26)	7.46 (6.15, 10.85)	0.040
ALB (g/L)	39.00 (36.00, 42.00)	40.00 (37.00, 42.00)	37.00 (34.00, 40.00)	<.001
ALT (U/L)	23.00 (16.00, 36.00)	24.00 (17.00, 36.00)	23.00 (16.00, 36.00)	0.382
AST (U/L)	38.00 (27.13, 63.75)	37.00 (27.00, 63.50)	41.00 (28.50, 63.00)	0.130
TC (mmol/L)	4.02 (3.23, 4.90)	3.97 (3.17, 4.78)	4.22 (3.42, 5.32)	<.001
TG (mmol/L)	1.37 (0.94, 2.09)	1.33 (0.89, 2.01)	1.46 (1.09, 2.21)	0.002
HDL-C (mmol/L)	0.94 (0.78, 1.13)	0.95 (0.79, 1.15)	0.87 (0.75, 1.08)	0.002
LDL-C (mmol/L)	2.23 (1.65, 2.98)	2.19 (1.63, 2.90)	2.41 (1.67, 3.27)	0.005
hsTnI (pg/ml)	3349.10 (630.60, 29434.50)	3703.70 (630.80, 31285.25)	2383.50 (631.65, 19706.30)	0.308
NT-proBNP (pg/ml)	1047.50 (300.62, 3382.50)	888.00 (265.55, 2647.50)	1891.00 (523.40, 6931.50)	<.001
GRACE	118.00 (97.00, 149.75)	116.00 (94.00, 145.00)	132.00 (103.00, 161.00)	<.001
CALLY	0.95 (0.24, 3.60)	1.19 (0.30, 5.03)	0.59 (0.15, 1.43)	<.001
LVEF (%)	58.00 (48.25, 62.00)	58.00 (51.00, 63.00)	53.00 (42.00, 60.00)	<.001
Treatment, *n* (%)				
CABG	37 (3.66)	30 (3.91)	7 (2.88)	0.456
PCI	924 (91.49)	717 (93.48)	207 (85.19)	<0.001
Conservative Therapy	49 (4.85)	20 (2.61)	29 (11.93)	<0.001
Aspirin	864 (85.54)	673 (87.74)	191 (78.60)	<0.001
P2Y12 inhibitors	891 (88.22)	692 (90.22)	199 (81.89)	<0.001
Beta-blockers	645 (63.86)	505 (65.84)	140 (57.61)	0.020
ACEi/ARB/ARNI	617 (61.09)	484 (63.10)	133 (54.73)	0.020
Diuretics	312 (30.89)	220 (28.68)	92 (37.86)	0.007
SGLT2i	313 (30.99)	244 (31.81)	69 (28.40)	0.315

BMI, body mass index; SBP, systolic blood pressure; DBP, diastolic blood pressure; HR, heart rate; WBC, white blood cell; NEUT, neutrophil; LYMPH, lymphocyte; Hb, hemoglobin; HbA1c, hemoglobin A1c%; CRP, C-reactive protein; Crea, creatinine; Alb, albumin; ALT, alanine transaminase; AST, aspartate aminotransferase; TC, total cholesterol; TG, triglyceride; HDL-C, high-density lipoprotein cholesterol; LDL-C, low-density lipoprotein cholesterol; NT-proBNP, brain natriuretic peptide; LVEF, left ventricular ejection fraction; CABG, coronary artery bypass grafting; PCI, percutaneous coronary intervention.

### Baseline characteristics of patients grouped by CALLY levels

We used X-tile software to calculate the optimal cutoff value, which was found to be 2.80, and proceeded with grouping and further analysis. As shown in Table 2 that AMI patients were stratified into two groups: the low-level group (CALLY < 2.80, *n* = 707) and the high-level group (CALLY > 2.80, *n* = 303). Compared to the high-level group, patients in the low-level group exhibited significantly older age, heart rates, GRACE scores, and levels of Neut, CRP, Urea, Crea, Glu, ALB, AST, TC, HDL-C, hsTnI, and NT-proBNP (all *P* < 0.05). The prevalence of Hypertension and diabetes were also significantly higher in the low-level group (*P* < 0.05). Conversely, levels of SBP, DBP, Hb, LYMPH, ALB, Na^+^, Ca^2+^, TC, TG, HDL-C, and LVEF were significantly lower in the low-level group (all *P* < 0.05). In therapeutic regimens, the low-level group showed a lower proportion of PCI, with lower rates of aspirin and SGLT2i, while the use of diuretics was higher.

**Table 2:** Baseline characteristics of patients with AMI stratified by the median CALLY index.

Variables	Total (*n* = 1010)	Low CLAYY (*n* = 707)	High CLAYY (*n* = 303)	*P*
Age	67.50 (57.00, 75.00)	70.00 (59.00, 77.00)	63.00 (53.00, 72.00)	<.001
Male, *n* (%)	698 (69.11)	482 (68.18)	216 (71.29)	0.327
BMI (kg/m^2^)	23.44 (21.20, 24.60)	23.44 (21.20, 24.34)	23.44 (21.20, 24.61)	0.223
SBP (mmHg)	132.00 (117.00, 148.00)	132.00 (116.00, 148.00)	132.00 (118.00, 149.00)	0.696
DBP (mmHg)	77.00 (69.00, 89.00)	78.00 (69.00, 89.00)	77.00 (70.00, 89.00)	0.530
Heart rate (bpm)	78.00 (68.00, 90.00)	79.00 (70.00, 92.00)	75.00 (66.00, 86.00)	<.001
Smoking, *n* (%)	453 (44.85)	316 (44.70)	137 (45.21)	0.879
Drinking, *n* (%)	180 (17.82)	130 (18.39)	50 (16.50)	0.473
Hypertension, *n* (%)	601 (59.50)	436 (61.67)	165 (54.46)	0.032
Diabetes, *n* (%)	244 (24.16)	191 (27.02)	53 (17.49)	0.001
Stroke, *n* (%)	56 (5.54)	40 (5.66)	16 (5.28)	0.810
WBC (10^9^/L)	7.80 (6.18, 9.94)	7.81 (6.18, 10.30)	7.79 (6.21, 9.47)	0.350
NE (10^9^/L)	5.77 (4.10, 7.88)	6.00 (4.20, 8.30)	5.20 (4.03, 7.00)	<.001
LYMPH (10^9^/L)	1.33 (0.91, 1.80)	1.20 (0.82, 1.62)	1.60 (1.23, 2.16)	<.001
Hb (g/L)	134.00 (119.00, 147.00)	130.00 (114.00, 145.00)	140.00 (126.50, 151.50)	<.001
HbA1c (%)	5.90 (5.50, 6.70)	6.00 (5.50, 6.90)	5.90 (5.50, 6.60)	0.192
CRP (mg/L)	5.28 (1.52, 18.43)	11.79 (4.73, 30.75)	0.92 (0.47, 1.43)	<.001
K (mmol/L)	3.80 (3.50, 4.10)	3.80 (3.50, 4.10)	3.80 (3.50, 4.00)	0.584
Na (mmol/L)	139.00 (137.00, 141.00)	139.00 (136.00, 141.00)	140.00 (138.00, 141.00)	<.001
Cl (mmol/L)	107.00 (104.00, 109.00)	107.00 (104.00, 109.00)	107.00 (105.00, 109.00)	0.164
Ca (mmol/L)	2.20 (2.12, 2.28)	2.19 (2.10, 2.26)	2.23 (2.16, 2.31)	<.001
Urea (mmol/L)	5.66 (4.45, 7.39)	5.78 (4.49, 8.00)	5.55 (4.38, 6.79)	0.004
Crea (umol/L)	73.00 (62.00, 97.75)	75.00 (62.00, 105.00)	69.00 (61.00, 83.50)	<.001
Glu (mmol/L)	7.30 (5.90, 9.50)	7.40 (6.10, 10.02)	6.90 (5.50, 8.50)	<.001
ALB (g/L)	39.00 (36.00, 42.00)	38.00 (35.00, 41.00)	41.00 (38.00, 43.00)	<.001
ALT (U/L)	23.00 (16.00, 36.00)	24.00 (16.00, 37.00)	23.00 (17.00, 34.00)	0.263
AST (U/L)	38.00 (27.13, 63.75)	41.00 (28.00, 82.50)	34.00 (26.00, 47.50)	<.001
TC (mmol/L)	4.02 (3.23, 4.90)	3.99 (3.17, 4.82)	4.08 (3.39, 4.96)	0.022
TG (mmol/L)	1.37 (0.94, 2.09)	1.32 (0.94, 2.05)	1.44 (0.88, 2.26)	0.244
HDL-C (mmol/L)	0.94 (0.78, 1.13)	0.92 (0.77, 1.09)	0.99 (0.81, 1.18)	<.001
LDL-C, (mmol/L)	2.23 (1.65, 2.98)	2.24 (1.63, 3.00)	2.20 (1.69, 2.98)	0.772
hsTnI (pg/ml)	3349.10 (630.60, 29434.50)	4591.80 (745.25, 33171.35)	1329.70 (399.20, 16077.65)	<.001
NT-proBNP (pg/ml)	1047.50 (300.62, 3382.50)	1484.00 (484.50, 4446.50)	497.90 (149.60, 1209.00)	<.001
GRACE	118.00 (97.00, 149.75)	125.00 (102.00, 157.00)	104.00 (88.00, 128.00)	<.001
CALLY	0.95 (0.24, 3.60)	0.43 (0.13, 1.09)	7.00 (4.29, 13.60)	<.001
LVEF (%)	58.00 (48.25, 62.00)	56.00 (46.50, 62.00)	60.00 (55.00, 64.00)	<.001
Treatment, *n* (%)				
CABG	37 (3.66)	27 (3.82)	10 (3.30)	0.688
PCI	924 (91.49)	637 (90.10)	287 (94.72)	0.016
Conservative Therapy	49 (4.85)	43 (6.08)	6 (1.98)	0.005
Aspirin	864 (85.54)	592 (83.73)	272 (89.77)	0.012
P2Y12 inhibitors	891 (88.22)	615 (86.99)	276 (91.09)	0.064
Beta-blockers	645 (63.86)	440 (62.23)	205 (67.66)	0.100
ACEi/ARB/ARNI	617 (61.09)	428 (60.54)	189 (62.38)	0.583
Diuretics	312 (30.89)	254 (35.93)	58 (19.14)	<0.001
SGLT2i	313 (30.99)	235 (33.24)	78 (25.74)	0.018

BMI, body mass index; SBP, systolic blood pressure; DBP, diastolic blood pressure; HR, heart rate; WBC, white blood cell; NEUT, neutrophil; LYMPH, lymphocyte; Hb, hemoglobin; HbA1c, hemoglobin A1c%; CRP, C-reactive protein; Crea, creatinine; Alb, albumin; ALT, alanine transaminase; AST, aspartate aminotransferase; TC, total cholesterol; TG, triglyceride; HDL-C, high-density lipoprotein cholesterol; LDL-C, low-density lipoprotein cholesterol; NT-proBNP, brain natriuretic peptide; LVEF, left ventricular ejection fraction; CABG, coronary artery bypass grafting; PCI, percutaneous coronary intervention.

### Correlation between CALLY index and cardiovascular and cerebrovascular risk factors

Multivariate Cox proportional hazards model analysis showed that lower CALLY was a risk factor for MACCE in AMI patients, whether as a continuous variable (HR = 0.83, 95% CI: 0.78–0.88; *P* < 0.001) or a categorical variable (HR = 0.23, 95% CI: 0.15–0.35; *P* < 0.001). After adjusting for multiple models by separately incorporating gender, age, blood pressure, medication history, and laboratory indicators, lower CALLY remains significantly associated with the risk of MACCE in AMI patients. Details are shown in Table 3. Of course, we also analyzed the CALLY index by quartile and obtained the same results (Supplementary Table S2).

**Table 3:** Cox regression models for the relationship between the CALLY index and major adverse cardiovascular and cerebrovascular events.

Variables		Model 1	Model 2	Model 3	Model 4
		HR (95%CI)	HR (95%CI)	HR (95%CI)	HR (95%CI)
Continuous		0.83 (0.78∼0.88)	0.85 (0.80∼0.90)	0.86 (0.81∼0.91)	0.86 (0.81∼0.91)
Categorical	T1	Ref.	Ref.	Ref.	Ref.
	T2	0.23 (0.15∼0.35)	0.26 (0.17∼0.40)	0.27 (0.18∼0.41)	0.29 (0.19∼0.44)

Model 1: unadjusted for covariates.

Model 2: Adjust for age, gender, BMI, smoking; drinking; SBP; DBP; hypertension, diabetes, and stroke.

Model 3: Model 2 + P2Y12 receptor inhibitors, ACEi/ARB/ARNI, aspirin, statins, SGLT2i, beta-blockers, diuretics.

Model 4: Model3 + LVEF, Crea, ALT, AST, TC, TG, HDL-C, LDL-C, hsTnI.

### Kaplan–Meier analysis

Kaplan–Meier survival curves indicate a statistically significant difference in cumulative survival rates among AMI patients across CALLY subgroups (log-rank *P* < 0.05). Consistent with this finding, patients with low CALLY scores had a higher incidence of MACCE (HR = 0.229 (0.151–0.347), as illustrated in Figure 2.

**Figure 2:**
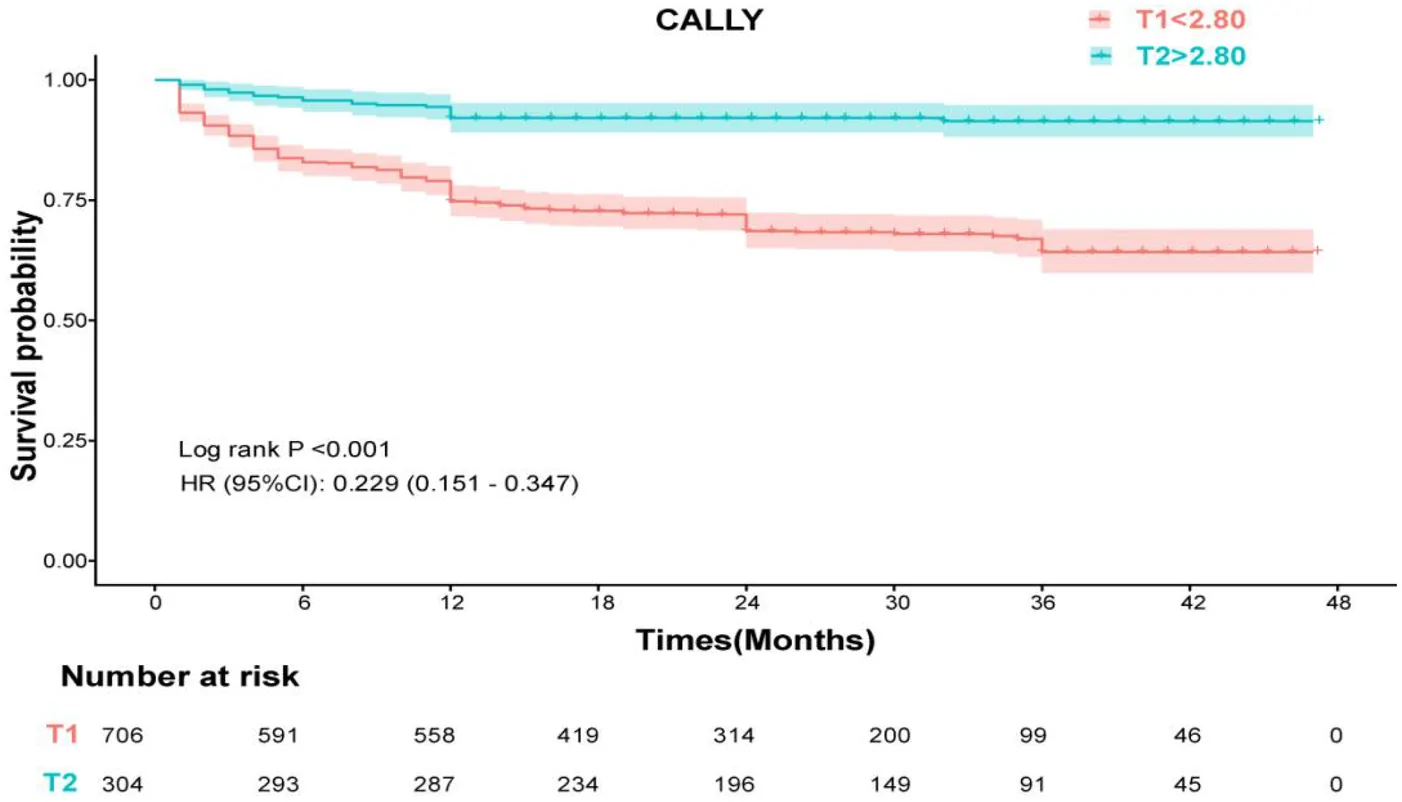
Kaplan–Meier curve illustrating the cumulative risk of cardiovascular and cerebrovascular events according to different CALLY index levels. CALLY index, C-reactive protein-albumin-lymphocyte index.

### Restricted cubic spline plot of CALLY index levels

The association of the CALLY index with the mortality outcomes was visualized in Figure 3 using restricted cubic spline (RCS). A non-linear and dose–response relationship between the CALLY index and the primary endpoint was observed (non-linear *P* = 0.003, *P* overall = 0.001). As the CALLY index increased, the risk of MACCE showed a downward trend. This decrease in risk was particularly pronounced at lower values of the CALLY index.

**Figure 3:**
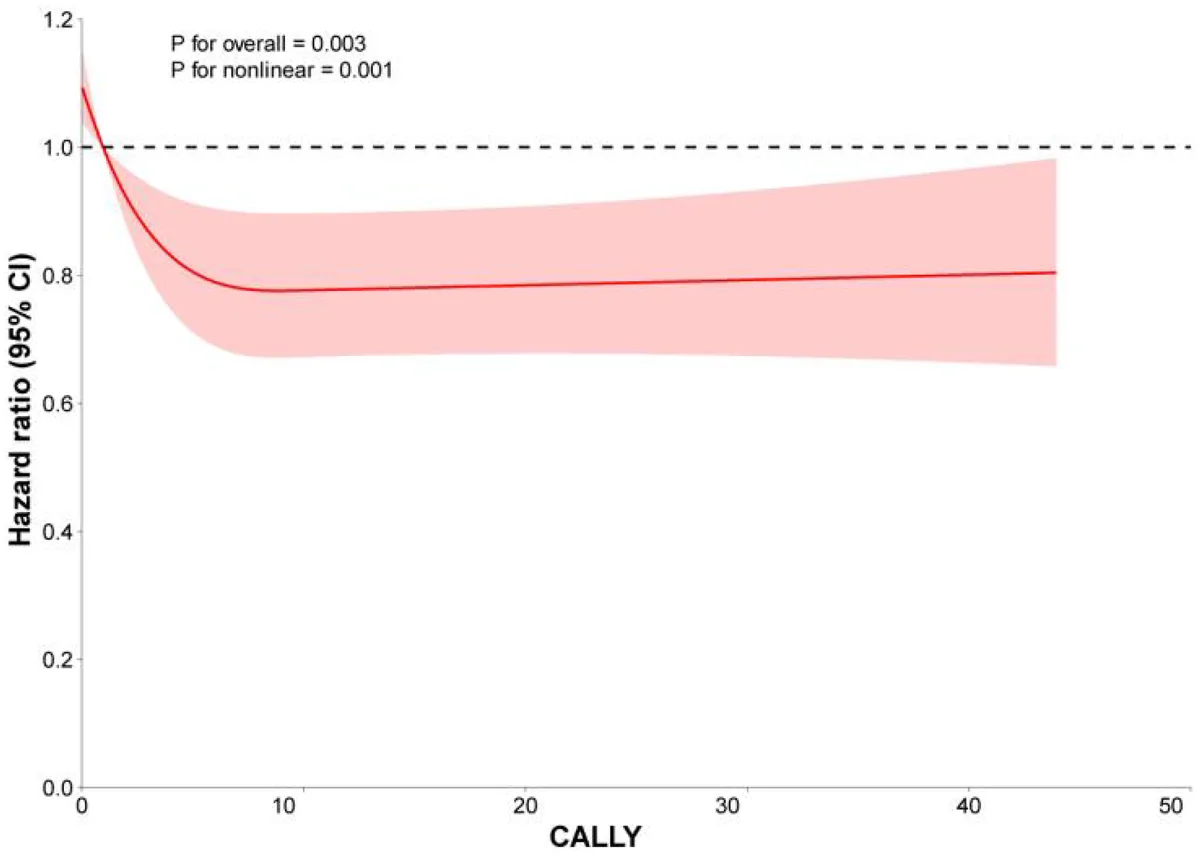
Restricted cubic spline plots based on CALLY.

### ROC curve analysis of CALLY index for MACCE in AMI patients

ROC curve analysis demonstrated (Figure 4) that the CALLY index possessed predictive value for the occurrence of MACCE in patients with AMI, and its predictive efficacy improved with the extension of the follow-up duration. At 6 and 12 months, the AUCs were 0.634 and 0.633, respectively; this indicated that the CALLY index might be more precise for long-term prognostic risk assessment. Notably, we also compared CALLY's predictive performance with that of other widely used metrics, including SII, PLR, PNI, and LCR, finding that CALLY yielded the highest predictive performance (Supplementary Figure S1).

**Figure 4:**
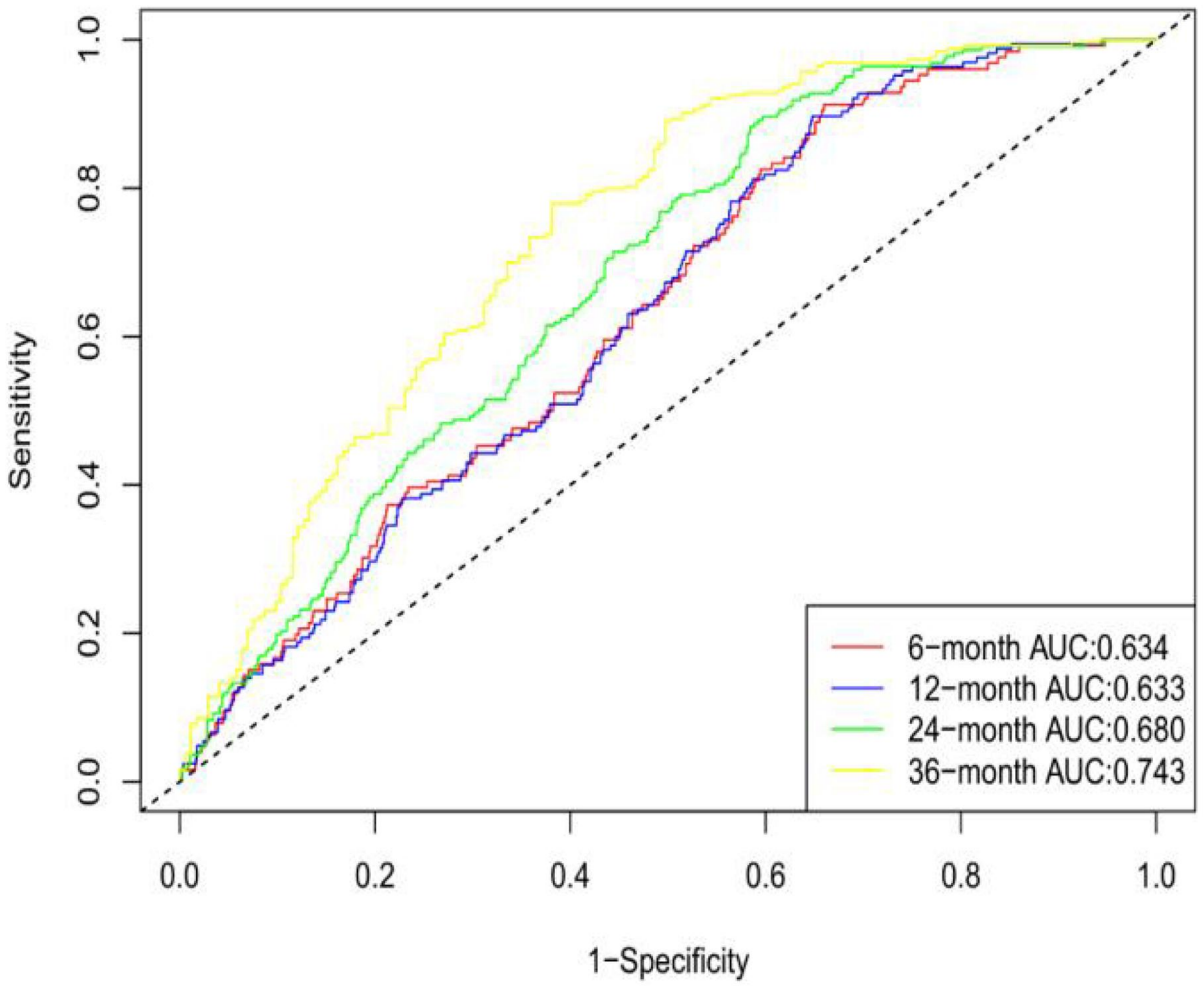
Time-dependent ROC curves for the prediction of MACCE.

### Subgroup analysis

Subgroup analysis was conducted to evaluate the robustness of the association between the CALLY index and the incidence of MACCE across various clinically relevant strata (Figure 5). An elevated CALLY index was associated with a reduced risk of MACCE in nearly all subgroups. The protective effect of the CALLY index did not differ significantly across subgroups defined by gender, history of hypertension, diabetes, or stroke, nor by type of myocardial infarction or age, with no evidence of interaction (all *P*-values for interaction >0.05). Therefore, the CALLY index serves as a broadly applicable and sensitive prognostic marker for patients with acute myocardial infarction, and its prognostic value appears robust across these common clinical characteristics.

**Figure 5:**
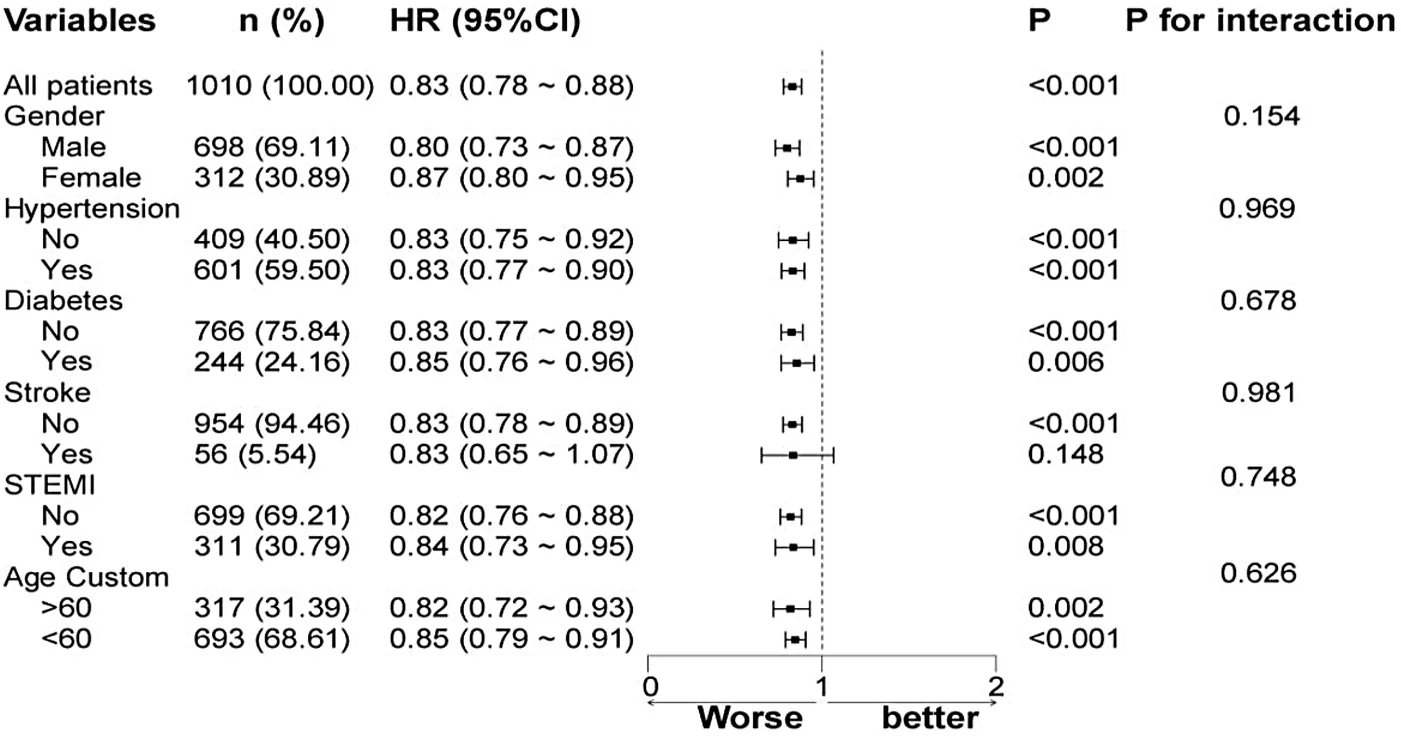
Subgroup and interaction analysis for the association between the CALLY index and MACCE.

### ROC curve analysis of the CALLY and GRACE scores for MACCE

AUC were used to examine the discriminative ability of CALLY index, GRACE score, and the combined model. As shown in Figure 6, ROC curve analysis showed that the combined model performed best. The AUC for CALLY index, GRACE score, and the combined model was 0.788 (95% CI: 0.742–0.835), 0.799 (95% CI: 0.737–0.861), and 0.853 (95% CI: 0.811–0.894), respectively. The DeLong test suggested a statistically significant difference between the combined model and CALLY index, or GRACE score in the ability to differentiate patients with MACCE (All *P* < 0.05). To further evaluate the improvement of CALLY on GRACE scores, we calculated the Net Receiver Operating Characteristic Improvement (NRI) and Integrated Discrimination Improvement (IDI) to quantify the incremental value of incorporating CALLY into the original model. The analysis yielded an IDI value of 0.017 (95% CI: 0.012–0.023, *P* < 0.001) and an NRI value of 0.366 (95% CI: 0.212–0.519, *P* < 0.001). Both metrics indicate a significant enhancement in risk classification capability. And the calibration plots demonstrate good calibration for the models (Figure 6B). The DCA curve shows that the new model provides a higher net benefit than the GRACE model alone for threshold probabilities (Figure 6C). The SHAP analysis suggests a new combination model, with CALLY ranking high in importance (Supplementary Figure S2).

**Figure 6:**
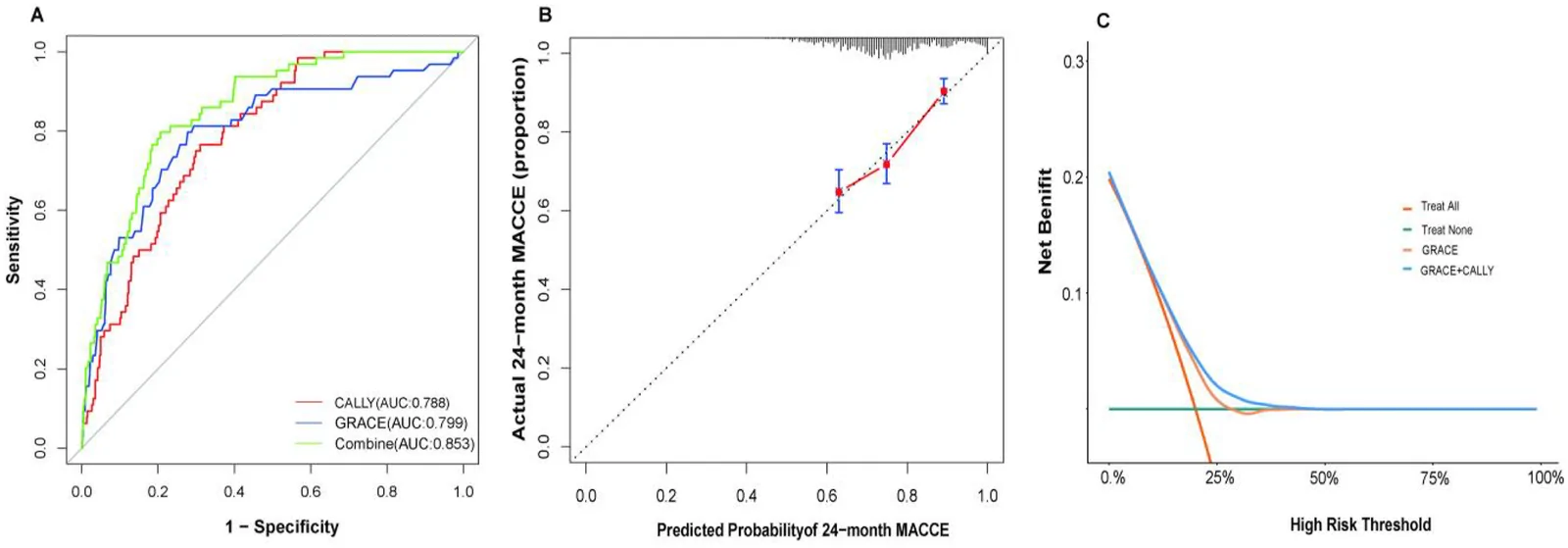
Evaluation of the combined model. **(A)** ROC curves for different indices and models; **(B)** calibration curves for the combined model; **(C)** DCA curves for different indices and models.

## Discussion

In this study, we evaluated the predictive value of the CALLY index for adverse outcomes in patients with AMI. The results showed that: (1) The CALLY index showed a significant negative correlation with adverse outcomes in AMI patients, and a low CALLY level served as an independent predictor of MACCE; (2) The CALLY index effectively addressed the limitations of the GRACE score regarding immune-inflammatory assessment, and incorporating it into the GRACE model significantly enhanced the model's predictive performance; (3) The study confirmed that the CALLY index demonstrated robust generalizability and sensitivity as a prognostic marker, and its predictive value remained consistent across different subgroups.

Inflammatory-immune mechanisms and malnutrition play a pivotal role in the progression of AMI, thereby influencing cardiac structural remodeling and the overall risk of sudden cardiac death. Consequently, early and precise assessment of the comprehensive “inflammation-immunity-nutrition” status is crucial for risk stratification and prognostic improvement in AMI patients (17). The CALLY index is a novel biomarker that integrates inflammatory response, nutritional status, and immune function, comprising C-reactive protein (CRP), serum albumin, and lymphocyte counts. The indicators underlying the CALLY index are readily available and cost-effective in clinical testing; the index effectively and simultaneously evaluates the inflammatory and nutritional status of patients, identifies high-risk populations with poor AMI prognosis at an early stage, and provides a reference for clinical decision-making and treatment planning (18).

As the core inflammatory component of the CALLY index, CRP played a pivotal role in determining the overall predictive performance of the index through its fluctuations. When CRP levels were elevated, the CALLY index decreased significantly even when albumin and lymphocyte counts remained within the normal range, reflecting a disruption in the systemic pro-inflammatory and anti-inflammatory balance. This characteristic confers high sensitivity in risk stratification upon the CALLY index, thereby enabling more accurate identification of high-risk patients (19). Primarily synthesized in the liver under the induction of cytokines such as interleukin-6 (IL-6) and interleukin-2 (IL-2) (20), CRP serves not only as a signal of inflammation but also directly binds to low-density lipoproteins and damaged cell membranes. This process activated the complement system and inhibited endothelial nitric oxide synthase (eNOS), leading to vascular endothelial dysfunction and plaque instability (21, 22). Previous large-scale cohort studies demonstrated that high CRP levels were independent predictors of all-cause mortality in patients with AMI and of short-term mortality following cardiogenic shock (23, 24). Consistent with the aforementioned literature, the results of the present study indicated that CRP levels in the MACCE group were significantly higher than those in the non-MACCE group (*P* < 0.001). This finding reaffirmed the central role of the inflammatory storm in the adverse prognosis of AMI. However, given the extreme complexity of the pathophysiological processes involved in AMI, a single CRP marker, while capable of reflecting the intensity of inflammation, failed to capture the concurrent states of metabolic consumption and immune paralysis in patients. Consequently, relying solely on CRP for risk stratification has inherent limitations (25).

Serum albumin serves not only as a classic marker for assessing nutritional status but also as a pivotal molecule in maintaining vascular homeostasis, thereby holding significant value for prognostic stratification in patients with AMI (26). Studies demonstrated that albumin, abundant in sulfhydryl groups, acted as the primary antioxidant and nitric oxide (NO) carrier in plasma, exerting vascular protective effects by scavenging oxygen free radicals and inhibiting platelet aggregation (27). During the acute phase of AMI, inflammatory stress inhibits albumin synthesis and accelerates catabolism (a negative acute-phase reaction); this secondary hypoalbuminemia signifies the weakening of the antioxidant defense barrier and abnormalities in blood viscosity (28, 29). Prior literature also supported this perspective: Liu et al. noted that hypoalbuminemia (<3.5 g/dL) was significantly associated with an approximate 40% increase in 30-day mortality among AMI patients (30); furthermore, a large-scale retrospective study involving 8,750 AMI patients confirmed that low admission albumin levels were independent predictors of increased all-cause mortality (31). Consistent with the aforementioned findings, the analysis in the present study revealed that baseline albumin levels in the MACCE group were significantly lower than those in the non-MACCE group (*P* < 0.001). This further suggests that low albumin levels reflect a dual pathological state characterized by “high metabolic consumption” and “high thrombotic risk.” However, albumin levels are susceptible to confounding influences from liver and kidney function, hemodynamic changes, and clinical interventions such as fluid resuscitation; consequently, the specificity of albumin as a solitary prognostic biomarker remains limited, underscoring the necessity for a combined multi-indicator assessment.

Lymphocytes represent a core component of the immune system and objectively reflect the body's immune defense and severity of physiological stress. Upon the onset of AMI, ischemic and necrotic cardiomyocytes stimulate the neurohumoral regulatory system and overactivate the cortisol-catecholamine axis. This process induces widespread lymphocyte apoptosis and redistribution, resulting in a sharp decline in lymphocyte counts (32, 33). The depletion of lymphocytes signifies not only the impairment of immune surveillance and repair functions but also the weakening of the antioxidant barrier. Consequently, the clearance of reactive oxygen species (ROS) is hindered, which further exacerbates myocardial oxidative stress injury (34). Our analysis reveals that lymphocyte counts are significantly and negatively correlated with all-cause mortality in patients with AMI (*Z* = −3.28, *P* = 0.001). Moreover, lymphocyte levels in the MACCE group were significantly lower than those in the non-MACCE group. This finding aligns closely with the study by Liu Yang et al., who identified that a lymphocyte count <1.2 × 10^9/L was an independent predictor of increased mortality (30). However, given that lymphocyte counts are confounded by infectious comorbidities and stress states, the specificity of this single indicator is limited. Therefore, combining it with inflammatory and nutritional indicators allows for a more robust and comprehensive assessment of the “inflammation-immune” pathological status in patients with AMI.

The onset and progression of AMI result from the combined effects of inflammation, immunity, and nutritional status. Reliance on a single inflammatory or immuno-nutritional indicator is often insufficient to fully reflect the patient's true pathophysiological status. Therefore, this study introduced the CALLY index, which was designed to leverage synergistic benefits through multi-dimensional integration. From the perspective of molecular mechanisms, AMI-induced myocardial ischemic necrosis activates NLRP3 or AIM2 inflammasomes, promoting the release of proinflammatory cytokines such as IL-1β and IL-18, which subsequently trigger a cascading systemic inflammatory response (35). This systemic stress results in characteristic changes in the components of the CALLY index: (1) Elevated CRP: As a core driver of the inflammatory storm, its levels directly reflect plaque instability and the degree of endothelial dysfunction; (2) Decreased albumin: The inflammation-mediated “negative acute phase reaction” inhibits albumin synthesis, leading to a compromise of the body's antioxidant defense barrier; and (3) Lymphocyte depletion: Ischemic stimulation activates the cortisol-catecholamine axis, inducing massive lymphocyte apoptosis and redistribution, which signals the impairment of immune surveillance and myocardial repair functions (36). Consequently, a reduction in the CALLY index serves as a robust indicator of a malignant state characterized by “high inflammatory load, low nutritional reserve, and immune defense failure.” Previous studies have initially confirmed the prognostic value of the CALLY index in the cardiovascular field. A retrospective cohort study involving 1,133 STEMI patients indicated that the CALLY index was an independent predictor of in-hospital mortality after PCI and served as an effective tool for risk stratification (37). Luo et al. found that a high CALLY index was significantly associated with reduced risks of all-cause and cardiovascular mortality in elderly patients (38). Furthermore, the index was negatively correlated with the length of hospital stay (*r* = −0.47, *P* < 0.001), suggesting that it reflected the degree of decompensation in heart failure patients (39). Additionally, He et al. confirmed in a population with heart failure with preserved ejection fraction (HFpEF) that a lower CALLY index (HR: 0.811, *P* = 0.001) was closely related to poor prognosis (40). Consistent with the aforementioned findings, this study further established the critical role of the CALLY index in evaluating the long-term prognosis of AMI. The data demonstrated that, whether analyzed as a continuous or categorical variable, all-cause and cardiovascular mortality rates in AMI patients significantly declined as the CALLY index increased. Multivariate Cox regression analysis showed that, compared with the low CALLY group, the risk of all-cause mortality in the high CALLY group decreased by approximately 46% (HR = 0.54, 95% CI: 0.42–0.70; *P* < 0.001). Kaplan–Meier survival analysis also confirmed that the 36-month cumulative survival rate of patients in the high CALLY group was significantly superior to that of the low CALLY group (HR = 0.539, 95% CI: 0.416–0.700; *P* < 0.001). In conclusion, as a composite biomarker integrating inflammation, immunity, and nutritional status, the CALLY index demonstrates excellent predictive efficacy and clinical applicability in AMI patients, facilitating the early identification of high-risk populations and the formulation of individualized intervention strategies.

The GRACE score was one of the commonly used tools for the clinical prognostic evaluation of ACS, and its predictive value was confirmed by multiple studies (41). In the present study, the GRACE score alone also demonstrated good predictive capability for MACCE events (AUC = 0.799, 95% CI: 0.737–0.861). However, the construction of the GRACE score lacked parameters reflecting key pathophysiological processes such as systemic inflammation, metabolic depletion, and coagulation activation. This limitation limits its capacity to fully capture the complex systemic pathological changes following AMI. Given the limitations of a single model, optimizing the GRACE score by integrating novel biomarkers emerged as a research hotspot in the cardiovascular field in recent years. Previous studies indicated that introducing indicators reflecting the body's overall reserve function significantly improved the model's sensitivity. For instance, some studies incorporated the Clinical Frailty Scale (CFS) and the Barthel Index (BI) into the evaluation system; by identifying frail patients with low physiological reserve, these studies successfully enhanced the discriminatory capacity of the GRACE score for 1-year mortality (42, 43). Another study involving 1,743 ACS patients focused on the nutritional dimension and found that malnutrition, as defined by the Nutritional Risk Index (NRI), not only independently predicted MACCE risk but also significantly improved prognostic prediction efficacy for the diabetic subgroup when incorporated into the GRACE model (44). These studies fully demonstrated that combining traditional “ischemia/hemodynamic assessment” with “systemic vulnerability assessment” was an effective approach to improving the accuracy of risk stratification. Aligned with the aforementioned concepts, this study innovatively combined the CALLY index—a composite indicator integrating “inflammation-nutrition-immunity”—with the GRACE score and validated its favorable clinical value. Statistical analysis showed that the combined model achieved a significant improvement in predictive efficacy for MACCE events, with the AUC rising from 0.799 for the GRACE score alone to 0.853 (95% CI: 0.811–0.894), and the difference was statistically significant. This improvement indicated that the CALLY index was not a mere repetition of the GRACE score but rather provided key information regarding metabolic status that the latter did not cover. The immune exhaustion and high inflammatory burden reflected by a low CALLY index effectively complements the limitations in pathophysiological mechanism assessment within the GRACE score. The increased predictive efficacy of the combined model suggested a potential synergistic effect at the pathophysiological level between the CALLY index and the GRACE score. The GRACE score focused on assessing cardiac pump failure and end-organ hypoperfusion, whereas the CALLY index focused on reflecting the body's systemic stress response and repair capability regarding acute injury. The combination of the two thereby facilitated a more precise identification of high-risk populations. Nevertheless, the specific mechanisms of action between the two remained not fully elucidated, and further basic and clinical research was required to explore the underlying mechanisms and verify the generalizability of this combined strategy across different ethnicities and clinical backgrounds.

This study has several limitations. First, due to the inherent constraints of a retrospective design, we were unable to dynamically measure CALLY levels during the patient follow-up period. Second, as a single-center study with a limited sample size, data bias could not be entirely avoided despite adjusting for multiple confounding factors. Third, due to the limited availability of clinical data, the prognostic differences between the CALLY index and other indicators, such as angiographic scoring systems, in patients with AMI were not investigated. Furthermore, prospective, multicenter studies are warranted to validate the applicability of this score across different populations and regions. Additionally, future research should explore whether interventions based on the CALLY index can improve patient outcomes.

## Conclusion

The results of this study indicate that a low CALLY index is significantly associated with poor prognosis in patients with AMI and serves as an independent predictor of MACCE. Furthermore, the CALLY index provides incremental predictive value to the GRACE score. The CALLY index shows promise as a practical and prospective biomarker for risk stratification and prognostic assessment in patients with AMI.

## Data Availability

The raw data supporting the conclusions of this article will be made available by the authors, without undue reservation.
