# Supplementary material for: Prognostic value of CALLY index in combination with the GRACE score among patients with acute myocardial infarction: a retrospective cohort study
Source: Front Cardiovasc Med. 2026 Sep 18;13:1858524. doi: 10.3389/fcvm.2026.1858524 (PMC13630654; doi:10.3389/fcvm.2026.1858524)
Supplement: Supplementary file 1 [file Datasheet1.docx]

Supplementary Material

**Prognostic value of CALLY index in combination with the GRACE score among patients with acute myocardial infarction: a retrospective cohort study**

Chuanglu Zhao^1†^，Rong Chen ^2†^，Ruirui Feng^1^，Xianlin Xu^1^, Hang Sun^1^, Xinwen Min^1^, Handong Yang^1^, Jishun Chen^1^*, Xiaolei Li^1^*

^1^Sinopharm Dongfeng General Hospital (Hubei Clinical Research Center of Hypertension), Hubei University of Medicine, Shiyan 442000, Hubei, China.

^2^Sinopharm Dongfeng Huaguo Hospital, 442000 Shiyan, Hubei, China

*Correspondence: Xiaolei Li, E-mail: [lixiaoleishz@163.com](mailto:lixiaoleishz@163.com)；Jishun Chen, E-mail:[376370276@qq.com](mailto:376370276@qq.com)

^†^Chuanglu Zhao and Rong Chen contributed equally to the writing of this article.

**Table S1. The number of cases for each type of MACCE**

| **Variables** | **Total** | **Male** | **Female** |
| --- | --- | --- | --- |
| MACCE | 243 | 155 | 88 |
| Cardiac deaths | 64 | 37 | 27 |
| Non-cardiac deaths | 14 | 10 | 4 |
| Non-fatal myocardial infarction | 33 | 20 | 13 |
| Target vessel revascularization | 78 | 50 | 28 |
| Heart failures | 33 | 28 | 5 |
| Non-fatal stroke | 21 | 10 | 11 |

**Table S2 Cox regression models for the relationship between the CALLY index and MACCE**

| Variables | | **Model 1** | **Model 2** | **Model 3** | **Model 4** |
| --- | --- | --- | --- | --- | --- |
|  |  | **HR(95%CI)** | **HR(95%CI)** | **HR(95%CI)** | **HR(95%CI)** |
| Categorical | T1 | Ref. | Ref. | Ref. | Ref. |
|  | T2 | 0.75 (0.54 ~ 1.03) | 0.77 (0.55 ~ 1.06) | 0.84 (0.60 ~ 1.16) | 0.87 (0.61 ~ 1.22) |
|  | T3 | 0.81 (0.59 ~ 1.11) | 0.90 (0.66 ~ 1.25) | 0.99 (0.71 ~ 1.38) | 0.99 (0.69 ~ 1.40) |
|  | T4 | 0.16 (0.10 ~ 0.28) | 0.20 (0.12 ~ 0.33) | 0.21 (0.13 ~ 0.36) | 0.23 (0.13 ~ 0.39) |

**Model 1:** unadjusted for covariates

**Model 2:** Adjust for age, gender, BMI, smoking; drinking; SBP; DBP; hypertension, diabetes, and stroke;

**Model 3:** Model 2+P2Y12 receptor inhibitors, ACEi/ARB/ARNI, aspirin, statins, SGLT2i, beta-blockers, diuretics;

**Model 4:** Model3+LVEF, Crea, ALT, AST, TC, TG, HDL-C , LDL-C, hsTnI.

**Fig.S1 Comparison of ROC Curves for Different Indices**

**Fig.S2 The SHAP Analysis**
